# Intrahepatic cholangiocarcinomas with IDH1/2 mutation-associated hypermethylation at selective genes and their clinicopathological features

**DOI:** 10.1038/s41598-020-72810-0

**Published:** 2020-09-25

**Authors:** Kyoungbun Lee, Young Seok Song, Yoonju Shin, Xianyu Wen, Younghoon Kim, Nam-Yun Cho, Jeong Mo Bae, Gyeong Hoon Kang

**Affiliations:** 1 Department of Pathology, Seoul National University College of Medicine, 103 Daehak-ro, Jongno-gu, Seoul, 03080 Korea; 2 Laboratory of Epigenetics, Cancer Research Institute, Seoul National University College of Medicine, Seoul, Korea

**Keywords:** DNA methylation, Bile duct cancer

## Abstract

Intrahepatic cholangiocarcinoma (ICC) is a rare but fatal tumor. The isocitrate dehydrogenase 1 and 2 (*IDH1*/*2*) genes are known to be mutated in ICC. *IDH1*/*2* mutations tend to be accompanied by enhanced hypermethylation at a subset of genomic loci. We sought to clarify the clinicopathological features, including prognostic value, of ICCs with *IDH1*/*2* mutation-associated hypermethylation at a subset of genes. The mutation status of *IDH1*/*2* and methylation status of 30 gene CpG island loci were analyzed in 172 cases of ICC using pyrosequencing and the MethyLight assay, respectively. The mutation status of *IDH1*/*2* was correlated with clinicopathological features and the DNA methylation status at 30 gene loci. Then, the clinicopathological characteristics were analyzed regarding three-tiered methylation statuses in genes showing *IDH1*/*2* mutation-associated methylation. *IDH1*/*2* mutations were found in 9.3% of ICCs, and *IDH1*/*2*-mutated tumors were associated with the histological subtype, including the bile ductular type and small duct type, and poor differentiation. Eight DNA methylation markers showed associations with *IDH1*/*2* mutations, and ICCs with > 5/8 methylated markers were associated with the bile ductular type or small duct type, absence of mucin production, absence of biliary intraepithelial neoplasia, and presence of chronic liver disease. > 5/8 methylated markers were an independent prognostic marker associated with better survival in both cancer-specific survival and recurrence-free survival. In summary, by analyzing the association between *IDH1*/*2* mutations and DNA methylation in individual genes, we developed a panel of DNA methylation markers that were significantly associated with *IDH1*/*2* mutations and were able to identify a subset of ICC with better clinical outcomes.

## Introduction

Approximately 2.8 × 10^7^ 5-methyl CpG dinucleotides are present in the genome, and the enzymes that generate and maintain methylation at the CpG dinucleotides are DNMT3A/B and DNMT1, respectively. The enzyme that demethylates 5-methyl CpG dinucleotides was first identified in 2009 by Rao and colleagues, who demonstrated that TET1 catalyzes 5-methylcytosine (5-mC) to 5-hydroxymethylcytosine (5-hmC)^1^. In 2011, hydroxyglutarate produced by mutant IDH1/2 was demonstrated to inhibit the catalytic activity of the TET family of 5-mC hydroxylases, which have ketoglutarate- and oxygen-dependent demethylation activity^2^. Zhang and colleagues further demonstrated that not only TET1 but also TET2 or 3 generates 5-hmC, 5-formyl cytosine, and 5-carboxyl cytosine through iterative oxidation of 5-mC^3^. *IDH1*/*2* mutations are mainly found in gliomas, acute myelogenous leukemia, and chondroid tumors but rarely found in epithelial malignancies except for cholangiocarcinomas^4^. Of the cholangiocarcinomas, *IDH1*/*2* mutations are found in intrahepatic cholangiocarcinomas (ICCs) and rarely, if ever, in perihilar or extrahepatic cholangiocarcinomas^5,6^.

ICC is a heterogeneous disease entity in terms of histology and is further classified into three histological subtypes, including the bile ductular (BD) type, small duct (SD) type, and large duct (LD) type^7–9^. The putative cell of origin of ICC varies according to the histological subtype^10–13^. ICC of the BD type might originate from hepatic progenitor cells at the canal of Herring or from biliary epithelial cells lining bile ductules, whereas ICC of the SD type might arise from biliary epithelial cells lining bile ductules or interlobular bile ducts, and ICC of the LD type might originate from epithelial cells lining the intrahepatic large bile ducts or peribiliary glands. According to the recently updated 2019 World Health Organization (WHO) classification, BD type was classified as a subtype of SD type^14^. Mutant IDHs produce 2-hydroxyglutarate and reduced nicotinamide adenine dinucleotide phosphate, both of which exert an effect on blocking the differentiation of hepatic progenitor cells toward hepatocytes but have no effect on bile duct differentiation^15^. The application of 2-hydroxyglutarate or induction of mutant IDHs in hepatic progenitor cells blocks differentiation toward hepatocytes^16^. The action of mutant IDHs in an analogous fashion has been suggested in leukemogenesis in which mutant IDHs disrupt hematopoietic stem cell differentiation^17^.

Although ICC has a relatively high frequency of *IDH1*/*2* mutations, the histomorphological features of ICC with *IDH1*/*2* mutations have not been well characterized. Kipp and colleagues first identified *IDH1*/*2* mutations in cholangiocarcinomas and a high frequency of *IDH1*/*2* mutations in ICCs compared with a low frequency of *IDH1*/*2* mutations in extrahepatic cholangiocarcinomas (28% vs. 7%, *P* = 0.030); they characterized the histological features of *IDH1*/*2*-mutated tumors as poor differentiation, clear cytoplasm, organoid arrangement of tumor cells, and relatively little desmoplasia^5^. However, it remains to be clarified whether ICCs with *IDH1*/*2* mutations show specific histological subtypes. Furthermore, controversy has been raised regarding the prognostic implications of *IDH1*/*2* mutations in patients.

In a previous study, we analyzed the methylation statuses of ICCs in 30 genes and found that frequently methylated genes were different between ICCs of the BD or SD type and ICCs of the LD type^18^. Of the 30 genes, six genes, including *DLEC1*, *RASSF1A*, *RIP3*, *SOCS3*, *PTGS2*, and *TNFRSF10C*, were more frequently methylated in ICCs of the BD type or SD type than in ICCs of the LD type. Of the six genes, five genes except for *TNFRSF10C* were more frequently methylated in hepatocellular carcinoma than in extrahepatic cholangiocarcinoma. Because it is known that DNA methylation profiles signifying the cell of origin are maintained during carcinogenesis, DNA methylation in the five genes might be postulated to signify a hepatic progenitor cell of origin in ICCs of the BD or SD type. In the present study, we analyzed 172 cases of ICC for their mutation statuses of *IDH1*/*2* and methylation statuses of 30 gene CpG island loci using the pyrosequencing assay and MethyLight assay, respectively. We sought to elucidate the clinicopathological features of ICCs with *IDH1*/*2* mutations, including the histological subtype and prognosis. By analyzing the association between *IDH1*/*2* mutations and DNA methylation at individual genes, we found that 8 DNA methylation markers, including *RIP3*, *DLEC1*, *PTGS2*, *MINT2*, *TNFRSF10C*, *RASSF1A*, *SOCS3*, and *ITF2*, were significantly associated with *IDH1*/*2* mutations. DNA methylation markers associated with *IDH1*/*2* mutations may be a signature of the IDH pathway. Because *IDH1* and *2* are not the only genes involved in the IDH pathway, the analysis of *IDH1*/*2* mutations will miss the identification of ICC cases that are not mutated at *IDH1*/*2* but are disturbed by alterations in other genes involved in the IDH pathway and disposed to hypermethylation at a specific set of the genes. We speculated that a panel of these eight DNA methylation markers were able to identify a subset of ICCs which are enriched in *IDH1*/*2* mutations. We found that the panel might help to identify a subset of ICC with characteristic clinicopathological features, including frequent *IDH1*/*2* mutations and better clinical outcomes.

## Material and methods

### Specimens

We collected archival tissue material from a consecutive series of ICC patients (n = 172) operated on at Seoul National University Hospital between January 2005 and December 2012. Pathological staging was determined according to the 7th version of the American Joint Committee on Cancer/Union for International Cancer Control (AJCC/UICC) tumor, node, metastasis (TNM) staging system. The determination of gross types, histological subtypes, and tumor grades were conducted as described previously^18^. Although the recently updated 2019 WHO classification classified ICCs into two main types, LD and SD, in a previous study, ICCs showed different clinical outcomes of a specific methylation marker between the BD type and SD type^18^. Thus, the present study retained three histologic types, BD, SD, and LD, rather than two main types, SD and LD. Through microscopic examination, we evaluated tissue slides for perineural invasion, lymphatic embolus, venous invasion, and the accompaniment of biliary disease, biliary intraepithelial neoplasia (BilIN), or chronic liver disease. This study was approved by the Institutional Review of Board of Seoul National University Hospital (IRB No. 1804-168-942). This study was conducted in compliance with the principles of the Declaration of Helsinki and its later amendments.

### DNA extraction

Tumor areas with the highest tumor purity and the most representative histology in the individual cases were marked through microscopic examination of the available tissue slides. The corresponding areas on unstained tissue slides were manually scraped with single edge-razor blades and collected into the microtubes. The collected tissues were incubated in lysis buffer containing 50 mM Tris, 1 mM EDTA (pH 8.0), 1% Tween-20, and proteinase K (3 mg/ml) at 55 °C for 24 h. The tissue lysates were subjected to heating at 95 °C for 30 min to inactivate proteinase K and denature genomic DNA. After centrifugation, the supernatants were transferred into newly labeled, clean microtubes. We did not purify DNA from the supernatants of tissue lysates but used tissue lysates directly for polymerase chain reaction (PCR) or bisulfite modification.

### Pyrosequencing assay

DNA from the tissue lysates was amplified by PCR with oligonucleotide primers specific to *IDH1* codon 132 and *IDH2* codon 172 (Supplementary Table 1). PCR was conducted in a total volume of 25 µL with the PyroMark PCR kit (Qiagen, Hilden, Germany) and final concentrations of 1X PyroMark PCR Mastermix, 1 × CollaLoad Concentrate, and 0.2 μM of each primer. Thermal cycling consisted of 45 cycles with denaturing (95 °C, 30 s), annealing (53 °C, 30 s), and elongation (72 °C, 30 s) steps, preceded by an initial denaturation step (95 °C, 10 min) and succeeded by a final extension step (72 °C, 10 min). Pyrosequencing for the detection of *IDH1* (codon 132) and *IDH2* (codon 172) mutations was performed using PyroGold reagents on the PyroMark Q24 pyrosequencer (Qiagen) according to the manufacturer’s instructions. Pyrogram outputs (Supplementary Fig. 1) were analyzed by PyroMark Q24 software (Qiagen).

**Table 1:** Clinicopathological features of intrahepatic cholangiocarcinoma with *IDH1*/*2* mutations.

	n	IDH1/2		*P*-value
		No mutation	mutation	
**Sex**				
M	121	110 (90.9%)	11 (9.1%)	0.999**
F	51	46 (90.2%)	5 (9.8%)	
**Age**				
<64 years	87	78 (89.7%)	9 (10.3%)	0.794
$\ge$64 years	85	78 (91.8%)	7 (8.2%)	
**Gross type**				
Mass forming	141	126 (89.4%)	15 (10.6%)	0.589^
Periductal infiltrative	8	8 (100%)	0	
Intraductal growing	18	17 (94.4%)	1 (5.6%)	
Mixed	5	5 (100%)	0	
**Differentiation**				
Well, moderate	117	110 (94.0%)	7 (6.0%)	0.031^#^
Poor	55	46 (83.6%)	9 (16.4%)	
**Histologic subtype**				
Bile ductular type	22	18 (81.8%)	4 (18.2%)	0.012^
Small duct type	68	58 (85.3%)	10 (14.7%)	
Large duct type	82	80 (97.6%)	2 (2.4%)	
**Intraglandular and/or extraglandular mucin production**				
Absent	78	68 (87.2%)	10 (12.8%)	0.190
Present	94	88 (93.6%)	6 (6.4%)	
**Lymphatic emboli**				
Absent	102	91 (89.2%)	11 (10.8%)	0.594
Present	70	65 (92.9%)	5 (7.1%)	
**Venous invasion**				
Absent	95	87 (91.6%)	8 (8.4%)	0.793
Present	77	69 (89.6%)	8 (10.4%)	
**Perineural invasion**				
Absent	118	104 (88.1%)	14 (11.9%)	0.098**
Present	54	52 (96.3%)	2 (3.7%)	
**Chronic liver disease**				
Absent	130	118 (90.8%)	12 (9.2%)	1.000**
Present	42	38 (90.5%)	4 (9.5%)	
BilIN*				
Absent	102	88 (86.3%)	14 (13.7%)	0.010**
Present	65	64 (98.5%)	1 (1.5%)	
**T category**				
T1	48	44 (91.7%)	4 (8.3%)	0.887^#^
T2	52	47 (90.4%)	5 (9.6%)	
T3	47	42 (89.4%)	5 (10.6%)	
T4	25	23 (92.0%)	2 (8.0%)	
**N category**				
pN0	133	119 (89.5%)	14 (10.5%)	0.312^#^
pN1	39	37 (94.9%)	2 (5.1%)	
**M category**				
pM0	161	145 (90.1%)	16 (8.9%)	0.278^#^
pM1	11	11 (100%)	0	
**TNM staging**				
Stage I	40	36 (90.0%)	4 (10.0%)	0.280^#^
Stage II	37	33 (89.2%)	4 (10.8%)	
Stage III	30	25 (83.3%)	5 (16.7%)	
Stage IVA	54	51 (94.4%)	3 (5.6%)	
Stage IVB	11	11 (100%)	0	

*Biliary intraepithelial neoplasia; 5 cases could not be assessed for biliary intraepithelial neoplasia.

**Fisher’s exact test,

^Kruskal–Wallis test,

^#^Wilcoxon’s rank-sum test.

**Figure 1:**
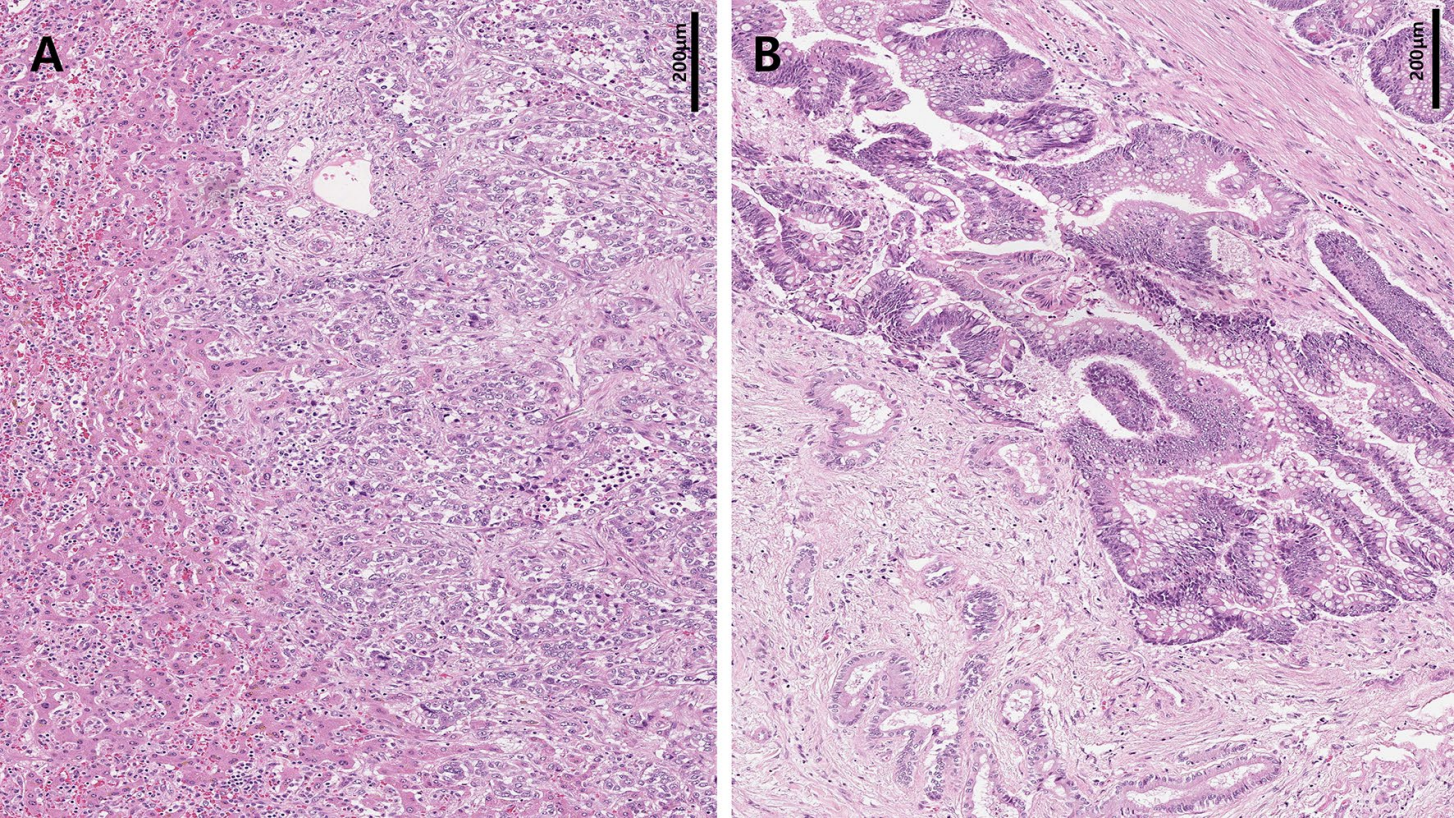
Histological features of *IDH1*/*2*-mutated intrahepatic cholangiocarcinomas (ICCs). ICCs with *IDH1*/*2* mutations were associated with a higher frequency of poor differentiation compared with ICCs without *IDH1*/*2* mutations. A case of ICC with *IDH1*/*2* mutations (**A**) and a case of ICC with no *IDH1*/*2* mutations (**B**).

### Bisulfite modification and the MethyLight assay

For the bisulfite modification of DNA samples, the EZ DNA Methylation Kit was used (Zymo Research, Orange, CA, USA). The modified DNA samples were subjected to *Alu*-based MethyLight control reaction to measure the amount of the modified DNA and were diluted with distilled water if the cross-threshold value went below 18. MethyLight assays were performed for the 30 CpG island loci as described previously^19^.

### Statistical analysis

IBM SPSS statistics (version 25) was used for the statistical analysis. Two-sided chi-square test was used for 2 × 2 contingency tables with minimal sample size > 5, whereas Fisher’s exact test (bidirectional) was used for 2 × 2 contingency tables with minimal sample size ≤ 5. Wilcoxon’s rank sum test and Kruskal–Wallis test were performed for ordinal variables and non-ordinal variables (contingency tables greater than 2 × 2), respectively. To identify whether the number of methylated genes was normally distributed in ICC tissue samples, normalization test was performed using the Shapiro–Wilk test, which revealed that the number of methylated genes were not normally distributed. Mean values of the number of methylated genes across two groups or across three or more groups were compared using the Mann–Whitney test and Kruskal–Wallis test, respectively. The cancer-specific survival (CSS) time was calculated from the date of surgery to the date of the death of the patient due to ICC. The recurrence-free survival (RFS) time was calculated from the date of surgery to the date of recurrence or death, whichever came first. CSS and RFS were compared between groups using the Kaplan–Meier method and log rank test. A Cox proportional hazards model with backward regression was used to investigate the association between the survival time of patients and one or more predictor variables.

### Ethical approval

This study was approved by the institutional review board of Seoul National University Hospital (IRB No. 1804-168-942). Under the condition of retrospective archival tissue collection and patient data anonymization, our study was exempted from the acquisition of informed consent from patients by the institutional review board of Seoul National University Hospital.

## Results

The overall study population included 172 patients who underwent surgical resection for ICC (Table 1). The median age at admission for operation was 63 years (range 38–80 years), and 70.3% of the patients were male. Of the 172 ICCs, the tumor growth patterns were the mass-forming type in 82.0%, periductal infiltrative type in 4.7%, intraductal growth type in 10.5%, and mixed type in 2.9%. The histological subtypes were BD type in 12.8%, SD type in 39.5%, and LD type in 47.7%. Chronic liver diseases and chronic biliary disease were found in the background liver of 24.4% and 7.6% of ICCs, respectively.

### *IDH1*/*2* mutations and their association with clinicopathological features

**Figure 2:**
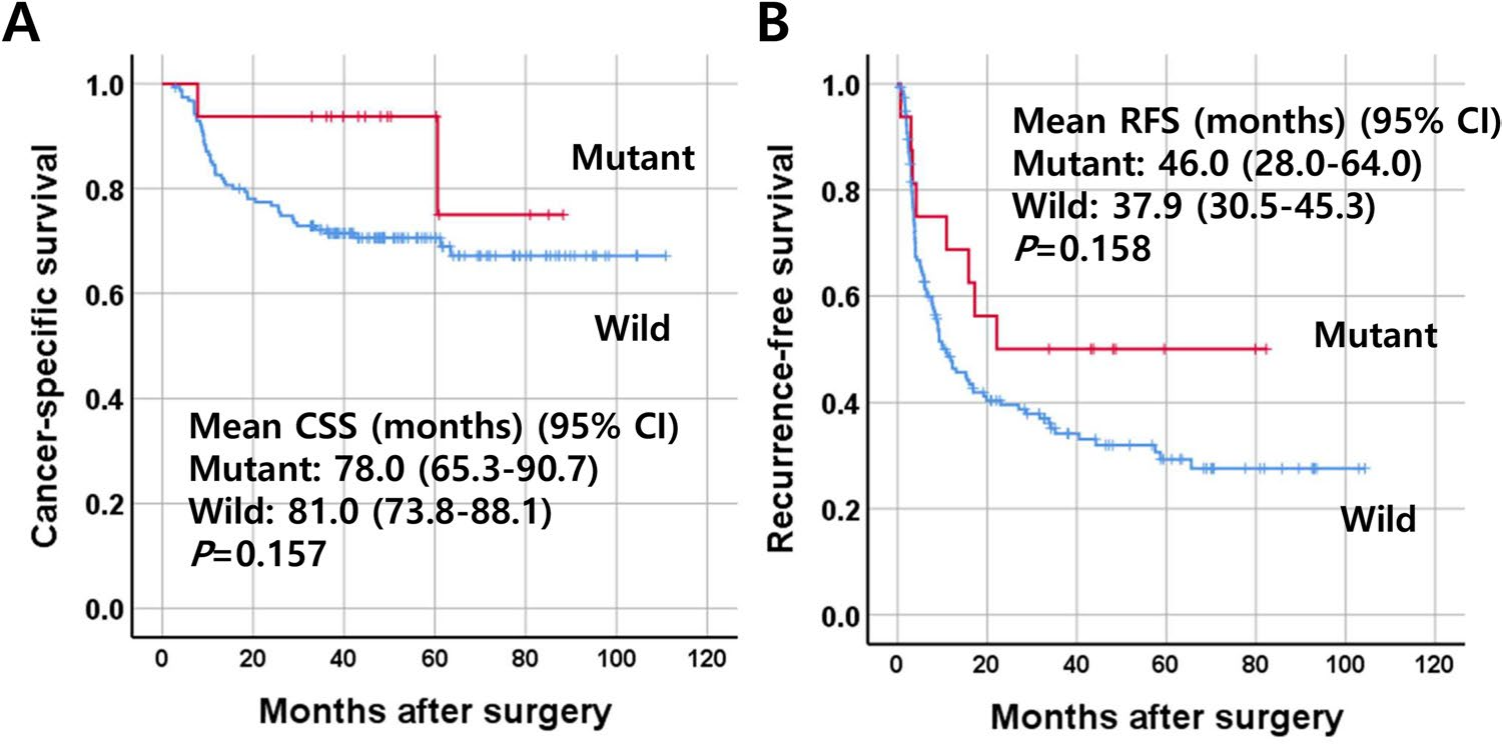
Survival curves of patients with intrahepatic cholangiocarcinoma according to the mutation status of IDH1/2. Cancer-specific survival (**A**) and recurrence-free survival (**B**).

A total of 16 patients (9.3%) showed either *IDH1* mutation (n = 12, 7.0%) or *IDH2* mutation (n = 4, 2.3%). *IDH1*/*2* mutations were associated with the histological subtypes: *IDH1*/*2* mutations were more frequent in the BD type and SD type (18.2% and 14.7%, respectively) than in the LD type (2.4%) (*P* = 0.003) (Table 1). *IDH1*/*2* mutations were associated with poor differentiation (*P* = 0.046) (Fig. 1). *IDH1*/*2* mutations were less frequent in ICCs with BilIN than in those without BilIN (1.5% vs. 13.7%, *P* = 0.010). Although marginally significant, ICCs with *IDH1*/*2* mutations tended to show less frequent perineural invasion (*P* = 0.098). In the survival analysis, ICCs with *IDH1*/*2* mutations tended to show better clinical outcomes in both CSS and RFS, which were statistically insignificant (Fig. 2).

### *IDH1/2* mutation-associated methylation markers and their usefulness for the identification of a subset of ICC with characteristic clinicopathological features

In a previous study, we analyzed the methylation statuses of ICC and normal duct tissues in the CpG island loci of 105 genes using the MethyLight assay and found 30 individual genes to be methylated in ICC tissues by more than 15% over normal duct tissues; these were regarded as genes showing cancer-related methylation. In the present study, we analyzed the relationship between *IDH1*/*2* mutations and the methylation of the 30 genes and found that 8 genes showed increased methylation frequencies in association with *IDH1*/*2* mutations, including *RIP3*, *DLEC1*, *PTGS2*, *MINT2*, *TNFRSF10C*, *RASSF1A*, *SOCS3*, and *ITF2* (in an order of decreasing *P*-values) (Supplementary Fig. 2 and Supplementary Table 2). When the relationships between the number of methylated genes and the clinicopathological parameters of ICC were examined among these 8 genes, an increased number of methylated genes was associated with male sex, lower T stage, BD or SD type, the absence of intraglandular and/or extraglandular mucin production, the absence of perineural invasion, the absence of BilIN, the presence of chronic liver disease, the absence of nodal metastasis, and lower TNM stage (Table 2). When we analyzed the relationship between the number of methylated genes and *IDH1*/*2* mutation (Supplementary Table 3), ICCs with > and ≤ 5/8 methylated genes showed *IDH1*/*2* mutation at a frequency of 52.2% and 2.7%, respectively, Compared with low-methylated ICCs (≤ 5/8 methylated genes), high-methylated ICCs (> 5/8 methylated genes) were associated with histological subtype of the BD or SD type, the absence of intraglandular and/or extraglandular mucin production, the absence of BilIN, and venous invasion (Supplementary Table 4).

**Table 2:** Comparison of the number of methylated markers in the panel in relation to clinicopathological features.

	n	No. of methylated markers	Standard deviation	*P*-value*
**Sex**				
M	121	3.6	1.82	0.008
F	51	2.8	1.84	
**Age (years)**				
< 64	87	3.3	1.86	0.775
≥ 64	85	3.4	1.86	
**Gross type**				
Mass forming type	141	3.5	1.91	0.161
Periductal infiltrative type	8	2.1	1.25	
Intraductal growing type	18	3.3	1.53	
Mixed type	5	2.60	1.52	
**Subtype**				
Bile ductular type	22	4.5	1.68	<0.001
Small duct type	68	3.8	2.13	
Large duct type	82	2.8	1.41	
**Grade**				
Well to moderately differentiated	117	3.2	1.67	0.074
Poorly differentiated	55	3.8	2.14	
**Intraglandular and/or extraglandular mucin**				
Absent	78	3.8	2.05	0.012
Present	94	3.0	1. 60	
**Lymphatic invasion**				
Absent	102	3.5	1.88	0.178
Present	70	3.2	1.90	
**Vascular invasion**				
Absent	95	3.5	1.68	0.232
Present	77	3.3	2.06	
**Perineural invasion**				
Absent	118	3.7	1.87	0.001
Present	54	2.7	1.63	
**T stage**				
T1	48	3.8	1.60	0.013
T2	52	3.7	2.08	
T3	47	2.7	1.66	
T4	25	3.3	1.90	
**N stage**				
pN0	133	3.6	1.96	0.010
pN1	39	2.7	1.79	
**M stage**				
pM0	161	3.5	1.87	0.074
pM1	11	2.4	1.29	
**TNM stage**				
I	40	3.8	0.260	0.005
II	37	4.1	0.344	
III	30	3.0	0.294	
IVA	54	2.9	0.253	
IVB	11	2.4	0.388	
**BilIN**				
Absent	102	3.7	2.11	0.031
Present	60	2.9	1.24	
**Chronic liver disease**				
Absent	130	3.2	1.79	0.068
Present	42	3.9	1.98	
**Chronic biliary disease**				
Absent	159	3.4	1.86	0.391
Present	13	2.9	1.82

**Table 3:** Multivariate survival analysis.

		HR (95% CI)	*P*-value
**Cancer-specific survival**^**a**^			
Methylation status of the eight methylation markers			
Low methylation	149	Ref	
High methylation	23	0.30 (0.09–0.97)	0.040
**Recurrence-free survival**^**a**^			
Methylation status of the eight methylation markers			
Low methylation	149	Ref	
High methylation	23	0.35 (0.17–0.72)	0.005

^a^Adjusted for gross type, histological type, differentiation grade, lymphatic emboli, venous invasion, perineural invasion, T stage, N stage, M stage, and *IDH1*/*2* mutation status.

### Survival analysis according to the methylation statuses of eight methylation markers

**Figure 3:**
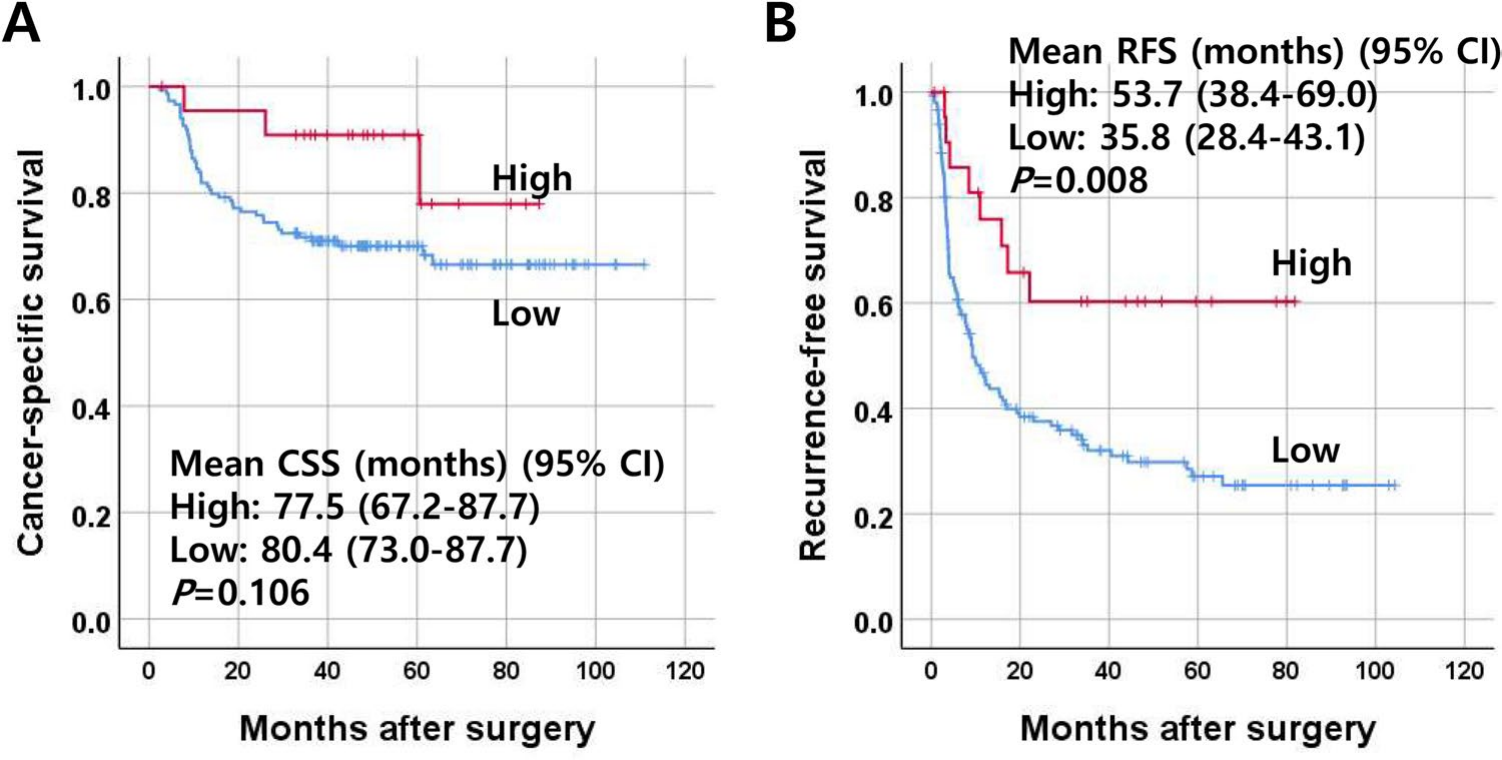
Survival curves of patients with intrahepatic cholangiocarcinoma (n = 172) according to the methylation status of the eight DNA methylation markers. ICCs were classified into two subgroups according to the number of methylated markers, including low-(≤ 5/8 methylated markers), and high-(> 5/8 methylated markers) methylation. Cancer-specific survival (**A**) and recurrence-free survival (**B**).

Kaplan–Meier survival curve analysis revealed that the recurrence-free survival rate of ICCs with high-methylation status (> 5/8 methylated markers in the marker panel) was significantly higher than that of ICCs with low- methylation status. No significant difference was noted in the cancer-specific survival rate of ICCs according to the methylation status in the marker panel (Fig. 3A, B). When we performed multivariate analysis, high methylation status (> 5/8 methylated genes) was found to be an independent prognostic parameter for better survival in terms of CSS and RFS (Table 3). When we performed subgroup analysis, the prognostic implications of the high-methylation status (> 5/8 methylated markers) in the marker panel were different depending on *IDH1*/*2* mutation status, a significant association with survival was found in ICCs with no *IDH1*/*2* mutation. For RFS, ICCs with high methylation showed better survival than ICCs with low methylation (Supplementary Fig. 3A–D).

## Discussion

In the present study, we analyzed the mutation status of ICCs in codon 132 of *IDH1* and codon 172 of *IDH2* using a pyrosequencing assay and found that *IDH1* or *2* was mutated in 9.3% of ICCs. When we explored cBioPortal for *IDH1*/*2* mutations in biliary tract cancers (n = 182), including gallbladder cancer, *IDH1* and *IDH2* mutations were found to be restricted to codon 132 and codon 172, respectively^20,21^. The frequency of *IDH1*/*2* mutations was lower in our study than in Western studies of ICCs. In Jiao et al.’s study in which a series of ICC specimens (n = 32) were sequenced for their exomes, 6 ICCs (19%) showed *IDH1*/*2* mutations^22^. In Kwong et al.’s study in which 32 ICC specimens were sequenced, *IDH1*/*2* mutations were found in 7 ICCs (22%)^23^. In a study using the SNaPshot genotyping assay, 40 of 200 ICCs (20%) showed *IDH1*/*2* mutations^24^. However, in a study that performed a comparative analysis to ICCs from Asian patients and compared the mutation frequency of *IDH1*/*2* between liver fluke-related and nonrelated ICCs, a significant difference was found in the mutation frequency of *IDH1*/*2* between liver fluke-related and nonrelated ICCs; the mutation frequency of *IDH1*/*2* was significantly higher in *Opisthorchis viverrini*-nonrelated ICCs (n = 27) than in *O. viverrini*-related ICCs (n = 62) (22.2% vs. 3.2%, *P* = 0.009)^25^. The mutation frequency of *IDH1*/*2* in Asian individuals with *O. viverrini*-nonrelated ICC was in agreement with the mutation frequency of *IDH1*/*2* in Western individuals with *O. viverrini*-nonrelated ICC. Although we could not obtain information regarding the infection status of *Clonorchis sinensis* in patients with ICC, Korea has geographical variation in terms of *C. sinensis* prevalence ranging from 2.1 to 31.3%^26^, and 9.5% of cholangiocarcinomas are estimated to be caused by chronic infection with *C. sinensis*^27^. In the endemic areas of Korea, 22.6% of cholangiocarcinoma cases were attributable to *C. sinensis* infection^27^. Thus, the lower rate of *IDH1*/*2* mutations in our ICC cases might be partly attributable to *C. sinensis* infection. Another attributing factor is related to the high proportion of the LD subtype in ICCs, and the LD subtype was less likely to contain *IDH1*/*2* mutations compared with the other subtypes of ICCs.

When we explored the relationship between *IDH1*/*2* mutations and clinicopathological features, we found that the histological subtype was significantly associated with *IDH1*/*2* mutations. *IDH1*/*2* mutations were mainly found in ICCs of the BD or SD subtype but rarely found in ICCs of the LD type. ICCs with *IDH1*/*2* mutations showed more frequent poor differentiation compared with ICCs with no *IDH1*/*2* mutations, which is in line with the findings of Kipp et al.’s study^5^. Kipp et al. analyzed the mutation statuses in *IDH1*/*2* of 94 cholangiocarcinomas and correlated them with clinicopathological features, revealing that ICCs with *IDH1*/*2* mutations were poorly differentiated. In Kipp et al.’s study, *IDH1*/*2*-mutated tumors were characterized by an organoid arrangement of tumor cells featured by an amphophilic cytoplasm and discrete cell borders or a compact arrangement of small tumor glands in stroma with relatively little desmoplasia. Although it is unclear whether such histological descriptions are compatible with the BD or SD histological subtypes, it is clear that such descriptions counter those of the LD subtype, which is characterized by mucin production, relatively large gland lumina, and desmoplastic stroma. However, in Borger et al.’s study, which found *IDH1*/*2* mutations in 9 of 40 ICCs, *IDH1*/*2*-mutated ICCs were all well to moderately differentiated, with tumor glands embedded within moderate to abundant stromal tissue^6^.

In the present study, we explored the relationship between *IDH1*/*2* mutations and the methylation of 30 genes and found that 8 genes showed associations between the methylation of individual genes and *IDH1*/*2* mutations. However, when we compared the number of methylated genes between ICCs with and without *IDH1*/*2* mutations, no significant difference was identified in the 30 examined genes (18.6 vs. 16.1, *P* = 0.136; Mann–Whitney U-test). These results indicate that *IDH1*/*2* mutations did not lead to the diffuse enhancement of promoter CpG island loci but instead to the selective enhancement of specific CpG island loci, which was evidenced in Kwong et al.’s study, which analyzed the genome-wide methylation statuses of 38 cases of cholangiocarcinoma using the Illumina 450 K Infinium bead assay^23^. Kwong et al. performed unsupervised clustering of cholangiocarcinoma cases using CpG sites that showed cancer-specific methylation changes, which generated four clusters. They found two distinct hypermethylation clusters in which *IDH1*/*2-*mutated tumors belonged to one cluster only. The cluster containing *IDH1*/*2*-mutated tumors showed a lower number of methylated CpG sites compared with the other hypermethylated cluster. Furthermore, the CpG sites that were frequently methylated in the cluster containing *IDH1*/*2*-mutated tumors were less frequently methylated in the other hypermethylated cluster.

In the present study, a panel of *IDH1*/*2* mutation-associated DNA methylation markers could identify a subset of highly methylated ICCs that is characterized by the BD or SD histological subtype, the absence of mucin production, the absence of BilIN, and better survival. Such clinicopathological features remind us of a molecular subclass enriched with *IDH1*/*2* mutations that was defined by integrated multiplatform clustering analysis (genome, epigenome, and transcriptome)^23,28^. Such a molecular subclass has also been suggested in the review paper of Sia et al., who proposed two distinct molecular classes of ICCs, including the proliferation and inflammation classes. The proliferation class contains a subtype with stem cell–like features, hypermethylation, and *IDH1*/*2* mutations^29^. However, integrated multiplatform clustering analysis is less likely to be adopted in clinical practice because of the requirement of fresh tissue samples and the high expense for the examination, which warrants the development of a more practical method to identify a subclass of ICCs enriched with *IDH1*/*2* mutations. Further study is needed to determine how much highly methylated ICC defined by the eight-marker panel of the present study coincides with the *IDH1*/*2* mutation-enriched molecular subtype defined by multiplatform analysis.

To validate our finding, on a different cohort, that the methylation status of eight genes could identify a subset of ICC with better clinical outcomes, we conducted Kaplan–Meier survival analysis in the ICC cohort (n = 35) of pancancer methylation data listed in the UCSC Xena browser^30^. Cut-off was set at beta-value of 0.15 and 0.2 to define methylation (Supplementary Fig. 4). Regardless of the cut-off value, no significant difference was identified in the survival rate between ICCs with > 5/8 methylated genes and ICCs with ≤ 5/8 methylated genes. The reason for which survival analysis result was not reproduced in the TCGA cohort might be related to the following: (1) the small sample size of subgroups in ICC cohort of the TCGA, which undermines the power of survival analysis. (2) The collection of ICC cases from a single high-volume hospital or from multiple hospitals over the world, including Brazil, Italy, Canada and USA, might induce bias in survival analysis because of the heterogeneity of clinical methods and surgical strategies in a multi-center study^23^. Clinical outcomes are more favorable in high-volume centers than in less experienced centers^31^. And, (3) Analytical technology was different between TCGA and the present study to assess methylation levels at eight genes. The Infinium technology determines methylation percentage at single CpGs, whereas the MethyLight technology assess the relative prevalence of a particular pattern of DNA methylation at multiple CpGs. A large-scaled study from a high-volume expert center is needed to validate our finding that the methylation status at eight genes determined by the MethyLight technology could identify a subset of ICCs with better clinical outcomes.

In conclusion, *IDH1*/*2* mutations were observed in 9.3% of ICCs, and most of them were observed in the mass-forming gross type, bile ductular or small duct histological types. Mutations in *IDH1*/*2* were not significantly associated with the prognosis of the patients. Although the *IDH1/2* mutations did not show prognostic value, ICCs with *IDH1*/*2* mutations exhibited a propensity toward better clinical outcomes. However, 8 gene CpG island loci were found to be significantly enhanced in methylation in association with *IDH1*/*2* mutations, and methylation at > 5/8 of these genes was found to be able to identify a subset of ICCs with distinct clinicopathological features and good prognosis. Future studies are needed to investigate whether the eight-methylation marker panel can identify *IDH1*/*2* mutation-enriched molecular subtypes defined by multiplatform analysis.

## Supplementary information

Supplementary file 1.

Supplementary file 2.

Supplementary file 3.

Supplementary file 4.

Supplementary file 5.

Supplementary file 6.

Supplementary file 7.

Supplementary file 8.

Supplementary file 9.
