# Supplementary material for: Intrahepatic cholangiocarcinomas with IDH1/2 mutation-associated hypermethylation at selective genes and their clinicopathological features
Source: Sci Rep. 2020 Sep 25;10:15820. doi: 10.1038/s41598-020-72810-0 (PMC7519101; doi:10.1038/s41598-020-72810-0)
Supplement: Supplementary file 1 [file 41598_2020_72810_MOESM1_ESM.docx]

Supplementary Figure 1. Representative pyrograms of *IDH1* and *2* mutations.

Supplementary Figure 2. The methylation heatmap of 30 DNA methylation markers for intrahepatic cholangiocarcinomas (n=172) with mutant or wild-type IDH1/2. Light brown (4≤PMR<20), brown (20≤PMR<50), and dark brown (PMR≥50).

Supplementary Figure 3. Kaplan-Meier survival curves of the subgroup with no *IDH1*/*2* mutation (A & B) and the subgroup with *IDH1*/*2* mutation (C & D). Survival curves of patients with intrahepatic cholangiocarcinoma (ICC) according to the methylation status of the eight DNA methylation markers. ICCs were classified into low- and high- methylation tumors. Cancer-specific survival (A & C) and recurrence-free survival (B & D).

Supplementary Figure 4. Kaplan-Meier survival analysis of TCGA cohort of intrahepatic cholangiocarcinoma (ICC) (n=35). Beta-value of 0.15 (A & B) or 0.2 (C & D) was set at cut-off value to define methylation-positive. Survival curves of patients with ICC according to the methylation status of the eight DNA methylation markers. ICCs were classified into low-methylation (≤5 markers) and high-methylation (≥6 markers) tumors. Overall survival (A & C) and progression-free survival (B & D).
