# Supplementary figures and images for: Intrahepatic cholangiocarcinomas with IDH1/2 mutation-associated hypermethylation at selective genes and their clinicopathological features
Source: Sci Rep. 2020 Sep 25;10:15820. doi: 10.1038/s41598-020-72810-0 (PMC7519101; doi:10.1038/s41598-020-72810-0)

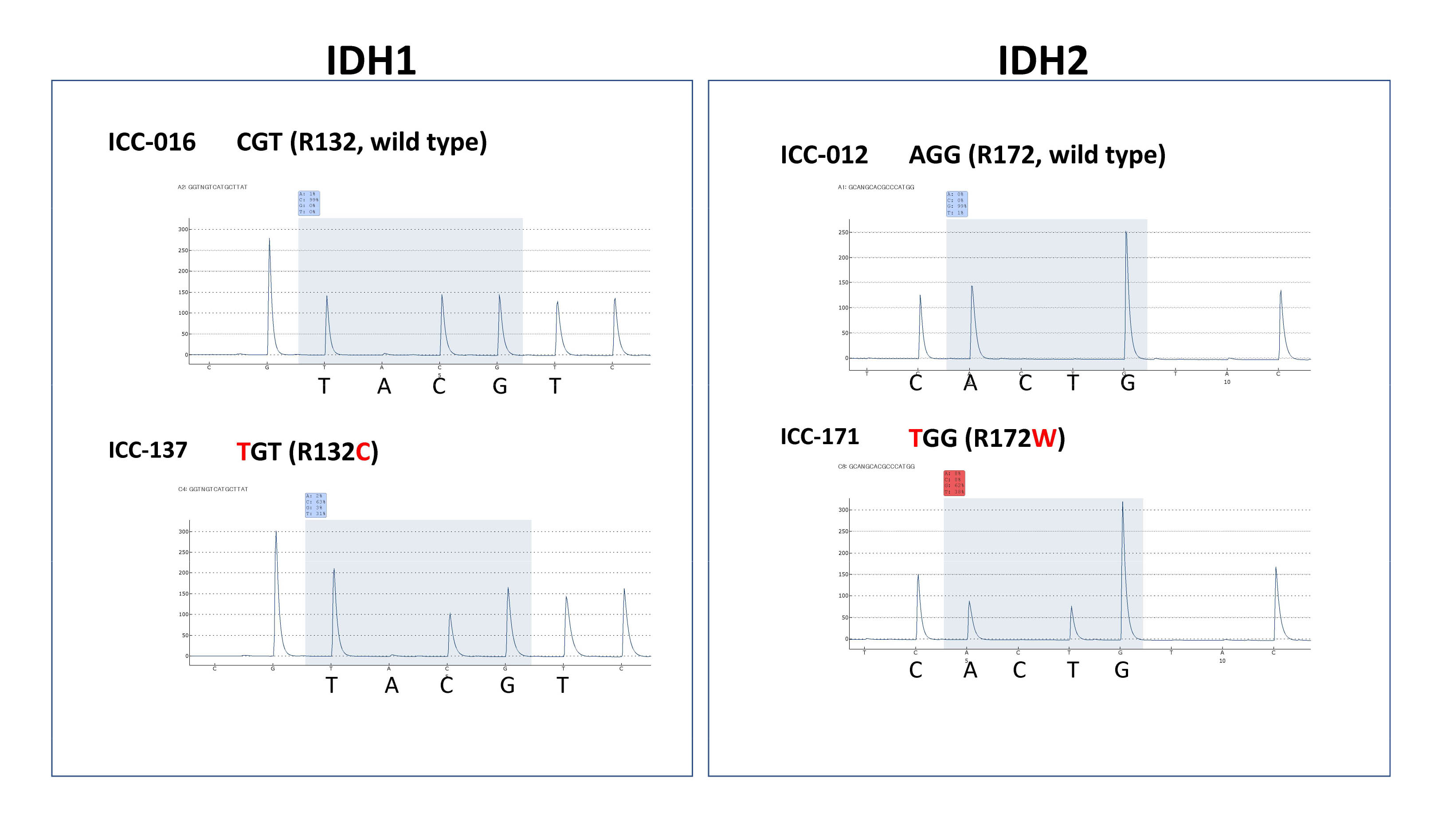

Supplement: Supplementary file 2 [file 41598_2020_72810_MOESM2_ESM.jpg]

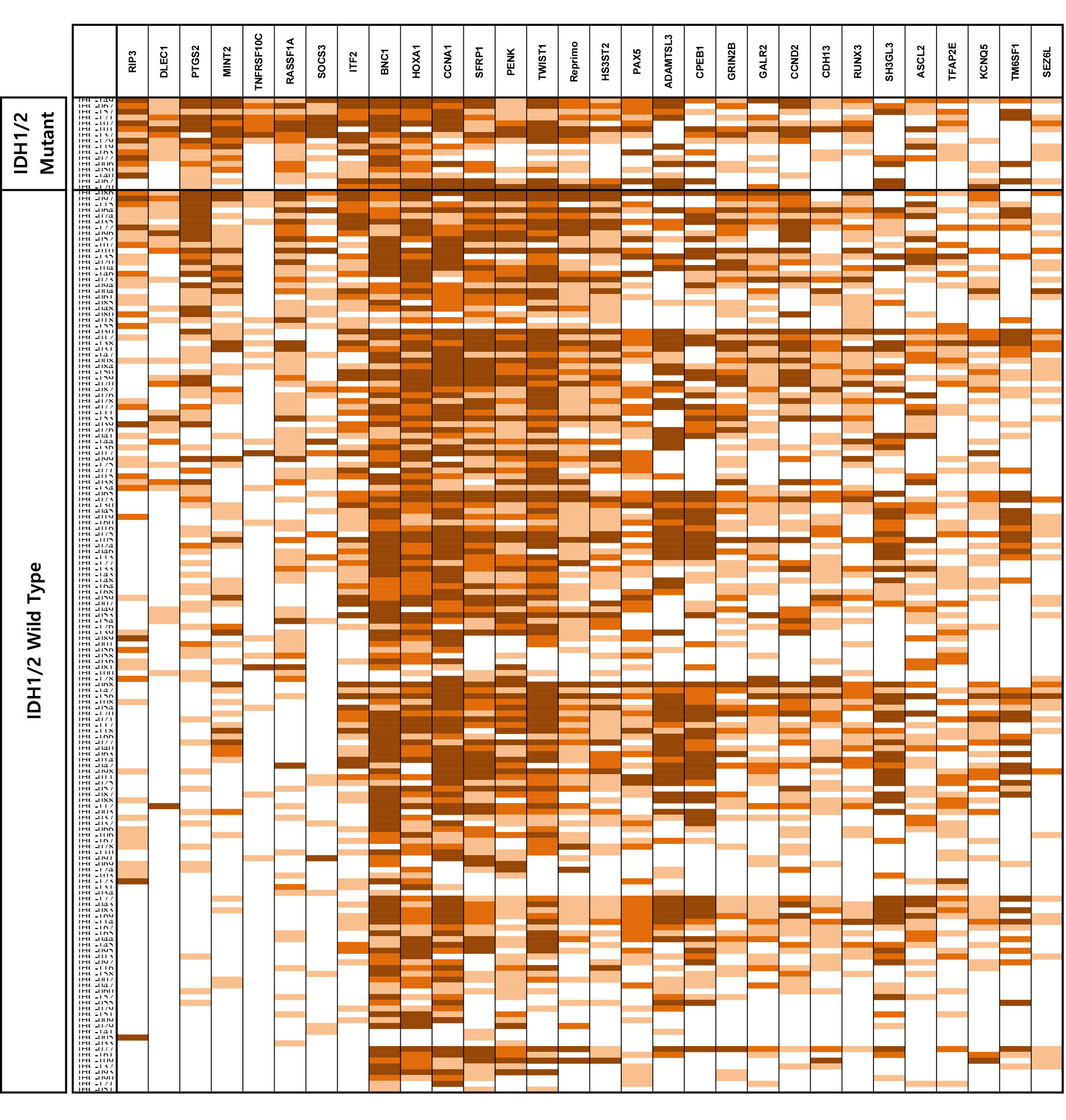

Supplement: Supplementary file 3 [file 41598_2020_72810_MOESM3_ESM.jpg]

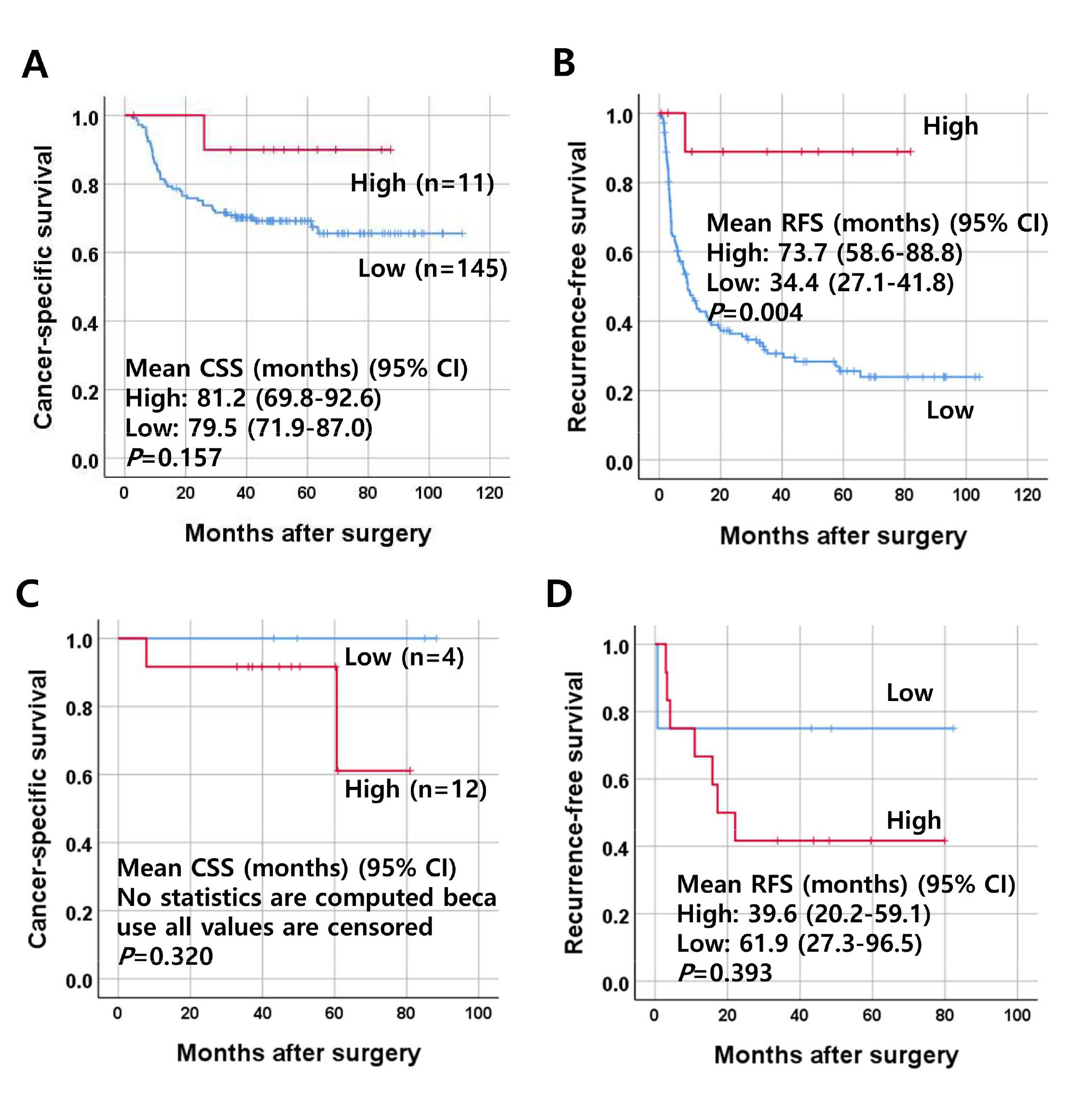

Supplement: Supplementary file 4 [file 41598_2020_72810_MOESM4_ESM.jpg]

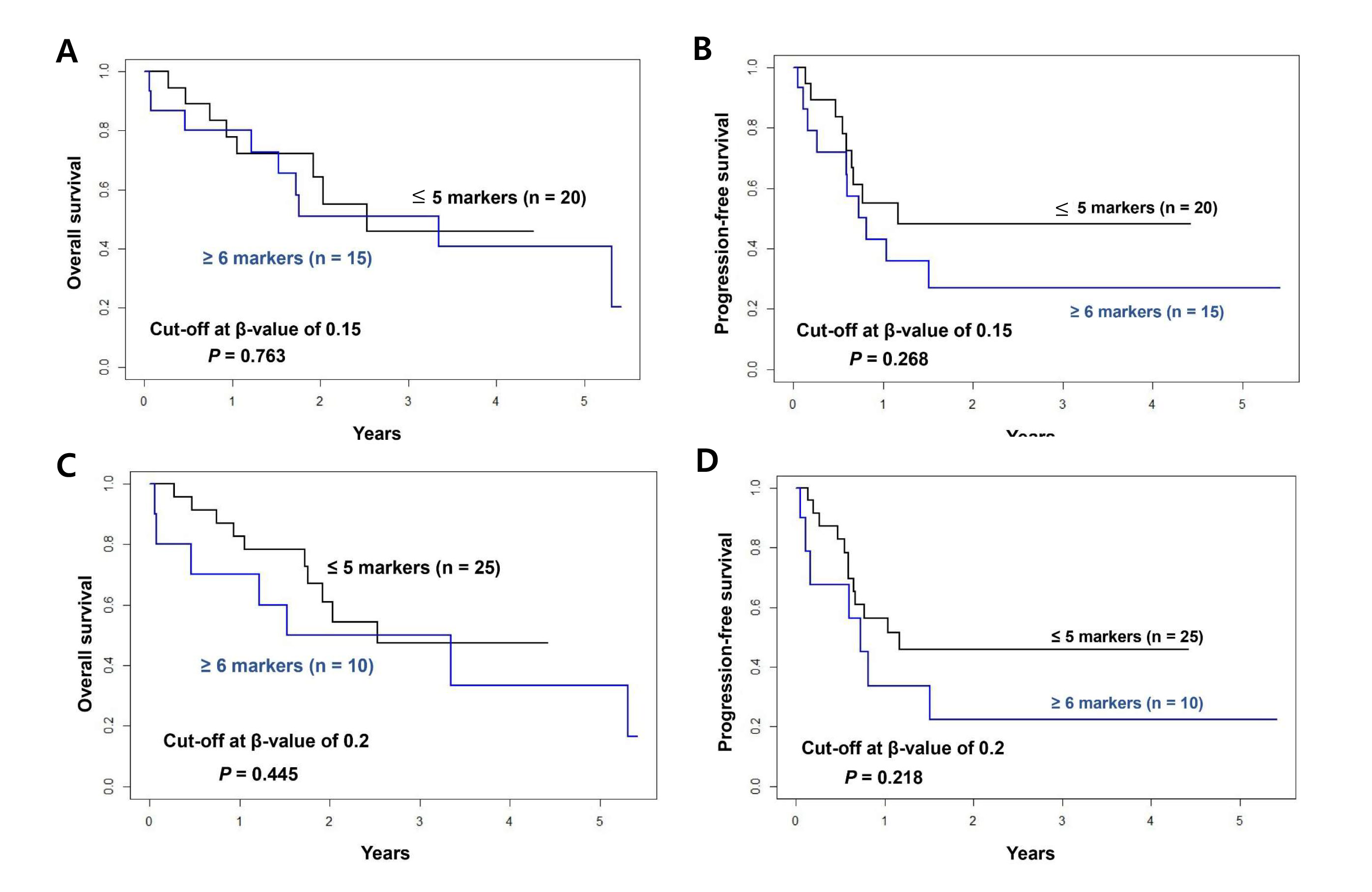

Supplement: Supplementary file 5 [file 41598_2020_72810_MOESM5_ESM.jpg]
