# Supplementary material for: Intrahepatic cholangiocarcinomas with IDH1/2 mutation-associated hypermethylation at selective genes and their clinicopathological features
Source: Sci Rep. 2020 Sep 25;10:15820. doi: 10.1038/s41598-020-72810-0 (PMC7519101; doi:10.1038/s41598-020-72810-0)
Supplement: Supplementary file 6 [file 41598_2020_72810_MOESM6_ESM.docx]

Supplementary Table 1. Oligonucleotide sequences of primers for pyrosequencing of the IDH1 and IDH2 genes.

| IDs | Primer name | Sequence (5’-3’) | Size (bp) |
| --- | --- | --- | --- |
| IDH1 | IDH1_132-PyF | GCTTGTGAGTGGATGGGTAAA | 75 |
|  | IDH1_132-PyR-Bt | Biotin-TTGCCAACATGACTTACTTGATC |  |
|  | IDH1_132-PyS2 | AAAACCTATCATCATAGGTC |  |
| IDH2 | IDH2_172-PyF | TCCGGGAGCCCATCATCT | 102 |
|  | IDH2_172-PyR-Bt | Biotin-CCTGGCCTACCTGGTCGC |  |
|  | IDH2_172-PyS | AGCCCATCACCATTG |  |
