# Supplementary material for: Intrahepatic cholangiocarcinomas with IDH1/2 mutation-associated hypermethylation at selective genes and their clinicopathological features
Source: Sci Rep. 2020 Sep 25;10:15820. doi: 10.1038/s41598-020-72810-0 (PMC7519101; doi:10.1038/s41598-020-72810-0)
Supplement: Supplementary file 7 [file 41598_2020_72810_MOESM7_ESM.docx]

Supplementary Table 2. Comparison of methylation frequency in 30 gene promoter CpG island loci between intrahepatic cholangiocarcinomas with wild-type and mutant *IDH1*/*2*

|  | No mutation (n=156) | Mutation (n=16) | *P*-value |  | No mutation (n=156) | Mutation (n=16) | *P*-value |
| --- | --- | --- | --- | --- | --- | --- | --- |
| *RIP3* | 52 (33.3%) | 14 (87.5%) | 3.369E-05 | *CPEB1* | 90 (57.7%) | 8 (50.0%) | 6.026E-01 |
| *DLEC1* | 29 (18.6%) | 11 (68.8%) | 5.766E-05 | *BNC1* | 144 (92.3%) | 16 (100%) | 6.063E-01 |
| *PTGS2* | 72 (46.2%) | 15 (93.8%) | 2.929E-04 | *GRIN2B* | 89 (57.1%) | 8 (50.0%) | 6.068E-01 |
| *MINT2* | 64 (41.0%) | 14 (87.5%) | 3.926E-04 | *HOXA1* | 136 (87.2%) | 15 (93.8%) | 6.964E-01 |
| *TNFRSF10C* | 19 (12.2%) | 8 (50.0%) | 7.343E-04 | *TM6SF1* | 58 (37.2%) | 5 (31.3%) | 7.879E-01 |
| *RASSF1A* | 81 (51.9%) | 15 (93.8%) | 1.027E-03 | *KCNQ5* | 69 (44.2%) | 6 (37.5%) | 7.923E-01 |
| *SOCS3* | 37 (23.7%) | 10 (62.5%) | 2.118E-03 | *ADAMTSL3* | 89 (57.1%) | 10 (62.5%) | 7.935E-01 |
| *ITF2* | 92 (59.0%) | 14 (87.5%) | 3.009E-02 | *CCND2* | 86 (55.1%) | 8 (50.0%) | 7.942E-01 |
| *SEZ6* | 49 (31.4%) | 8 (50.0%) | 1.645E-01 | *TFAP2EL* | 71 (45.5%) | 8 (50.0%) | 7.958E-01 |
| *PENK* | 130 (83.3%) | 11 (68.8%) | 1.712E-01 | *PAX5* | 91 (58.3%) | 10 (62.5%) | 7.966E-01 |
| *CDH13* | 84 (53.8%) | 6 (37.5%) | 2.940E-01 | *ASCL2* | 75 (48.1%) | 7 (43.8%) | 7.977E-01 |
| *SH3GL3* | 82 (52.6%) | 6 (37.5%) | 2.998E-01 | *CDH13* | 81 (51.9%) | 8 (50.0%) | 1.000E+00 |
| *CCNA1* | 136 (87.2%) | 13 (81.3%) | 3.633E-01 | *RUNX3* | 81 (51.9%) | 8 (50.0%) | 1.000E+00 |
| *Reprimo* | 109 (69.9%) | 13 (81.3%) | 4.030E-01 | *SFRP1* | 129 (82.7%) | 13 (81.3%) | 1.000E+00 |
| *HS3ST2* | 106 (67.9%) | 9 (56.3%) | 4.054E-01 | *GALR2* | 86 (90.5%) | 9 (9.5%) | 1.000E+00 |
