# Supplementary material for: Intrahepatic cholangiocarcinomas with IDH1/2 mutation-associated hypermethylation at selective genes and their clinicopathological features
Source: Sci Rep. 2020 Sep 25;10:15820. doi: 10.1038/s41598-020-72810-0 (PMC7519101; doi:10.1038/s41598-020-72810-0)
Supplement: Supplementary file 8 [file 41598_2020_72810_MOESM8_ESM.docx]

Supplementary Table 3. Frequency of IDH1/2 mutation in intrahepatic cholangiocarcinoma according to the number of methylated genes

| No. of methylated genes | IDH1/2 | |
| --- | --- | --- |
|  | Wild | Mutation |
| 0 | 5 | 0 |
| 1 | 22 | 0 |
| 2 | 34 (97.1%) | 1 (2.9%) |
| 3 | 36 | 0 |
| 4 | 25 | 0 |
| 5 | 23 (88.5%) | 3 (11.5%) |
| 6 | 5 (45.5%) | 6 (54.5%) |
| 7 | 6 (66.7%0 | 3 (33.3%) |
| 8 | 0 | 3 |
