# Supplementary material for: Intrahepatic cholangiocarcinomas with IDH1/2 mutation-associated hypermethylation at selective genes and their clinicopathological features
Source: Sci Rep. 2020 Sep 25;10:15820. doi: 10.1038/s41598-020-72810-0 (PMC7519101; doi:10.1038/s41598-020-72810-0)
Supplement: Supplementary file 9 [file 41598_2020_72810_MOESM9_ESM.docx]

Supplementary Table 4. Clinicopathological features of intrahepatic cholangiocarcinoma according to the methylation status of the eight genes

|  |  |  | No. of methylated markers | | *P*-value |
| --- | --- | --- | --- | --- | --- |
|  |  | n | 0-5 | 6-8 |  |
| Sex | M | 121 | 102 (84.3%) | 19 (15.7%) | 0.167* |
|  | F | 51 | 47 (92.2%) | 4 (7.8%) |  |
| Age | <64 years | 87 | 76 (87.4%) | 11 (12.6%) | 0.776 |
|  | $\geq$64 years | 85 | 73 (85.9%) | 12 (14.1%) |  |
| Gross type | Mass forming | 141 | 119 (84.4%) | 22 (15.6%) | 0.316** |
|  | Periductal infiltrative | 8 | 8 (100%) | 0 |  |
|  | Intraductal growing | 18 | 17 (94.4%) | 1 (5.6%) |  |
|  | Mixed | 5 | 5 (100%) | 0 |  |
| Histologic | Bile ductular type | 22 | 17 (77.3%) | 5 (22.7%) | <0.001** |
| subtype | Small duct type | 68 | 52 (76.5%) | 16 (23.5%) |  |
|  | Large duct type | 82 | 80 (97.6%) | 2 (2.4%) |  |
| Differentiation | Well, moderate | 117 | 107 (91.5%) | 10 (8.5%) | 0.007 |
|  | Poor | 55 | 42 (76.4%) | 13 (23.6%) |  |
| Intra- and/or extraglandular | Absent | 78 | 60 (76.9%) | 18 (23.1%) | 0.001* |
| mucin production | Present | 94 | 89 (94.7%) | 5 (5.3%) |  |
| Lymphatic | Absent | 102 | 89 (87.3%) | 13 (12.8%) | 0.771 |
| emboli | Present | 70 | 60 (85.7%) | 10 (14.3%) |  |
| Venous | Absent | 95 | 87 (91.6%) | 8 (8.4%) | 0.034 |
| invasion | Present | 77 | 62 (80.5%) | 15 (19.5%) |  |
| Perineural | Absent | 118 | 99 (83.9%) | 19 (16.1%) | 0.120* |
| invasion | Present | 54 | 50 (92.6%) | 4 (7.4%) |  |
| Chronic liver | Absent | 130 | 116 (89.2%) | 14 (10.8%) | 0.078 |
| disease | Present | 42 | 33 (78.6%) | 9 (21.4%) |  |
| Biliary intraepithelial neoplasia* | Absent | 102 | 81 (79.4%) | 21 (20.6%) | 0.002* |
|  | Present | 65 | 64 (98.5%) | 1 (1.5%) |  |
| T category | T1 | 48 | 45 (93.8%) | 3 (6.3%) | 0.447^#^ |
|  | T2 | 52 | 41 (78.8%) | 11 (21.2%) |  |
|  | T3 | 47 | 42 (89.4%) | 5 (10.6%) |  |
|  | T4 | 25 | 21 (84.0%) | 4 (16.0%) |  |
| N category | pN0 | 133 | 114 (85.7%) | 19 (14.3%) | 0.520^*^ |
|  | pN1 | 39 | 35 (89.7%) | 4 (10.3) |  |
| M category | pM0 | 161 | 138 (85.7%) | 23 (14.3%) | 0.183^*^ |
|  | pM1 | 11 | 11 (100%) | 0 |  |
| TNM staging | Stage I | 40 | 37 (92.5%) | 3 (7.5%) | 0.458^#^ |
|  | Stage II | 37 | 27 (73.0%) | 10 (27.0%) |  |
|  | Stage III | 30 | 26 (86.7%) | 4 (13.4%) |  |
|  | Stage IVA | 54 | 48 (88.9%) | 6 (11.1%) |  |
|  | Stage IVB | 11 | 11 (100%) | 0 |  |
| *IDH1*/*2* | Wild type | 156 | 84 (53.9%) | 11 (7.1%) | <0.001* |
|  | Mutant type | 16 | 3 (18.8%) | 12 (75.0%) |  |

*Fisher’s exact test

**Kruskal-Wallis test

#Wilcoxon’s rank sum test
